# The Need to Improve the Medical Subject Headings (MeSH) and the Excerpta Medica Tree (EMTREE) Thesauri to Perform Systematic Review on Oral Potentially Malignant Disorders

**DOI:** 10.1111/jop.13616

**Published:** 2025-03-09

**Authors:** Vito Carlo Alberto Caponio, Gennaro Musella, Mario Pérez‐Sayáns, Lorenzo Lo Muzio, Rui Amaral Mendes, Rosa María López‐Pintor

**Affiliations:** ^1^ Department of Clinical and Experimental Medicine University of Foggia Foggia Italy; ^2^ ORALMED Research Group, Department of Dental Clinical Specialties School of Dentistry, Complutense University Madrid Spain; ^3^ Oral Medicine, Oral Surgery and Implantology Unit (MedOralRes), Faculty of Medicine and Dentistry Santiago de Compostela University Santiago de Compostela Spain; ^4^ ORALRES Group Health Research Institute of Santiago de Compostela (FIDIS) Santiago de Compostela Spain; ^5^ Instituto de los materiales de Santiago de Compostela (iMATUS) Avenida do Mestre Mateo Santiago de Compostela Spain; ^6^ Department of Community Medicine, Information and Health Decision Sciences (MEDCIDS) Faculty of Medicine of the University of Porto Porto Portugal; ^7^ RISE‐Health PerMed Research Group, Faculty of Medicine of the University of Porto Porto Portugal; ^8^ RISE‐Laboratorio Associado LT2‐Clinical and Translational Research in Oncology, Faculty of Medicine of the University of Porto Porto Portugal; ^9^ Department of Oral and Maxillofacial Medicine and Diagnostic Sciences Case Western Reserve University Cleveland, OH USA

**Keywords:** medical informatics, meta‐analysis, mouth diseases, oral lichen planus, oral potentially malignant disorder, precancerous conditions

## Abstract

**Background:**

Despite recent advancements in the understanding and classification of oral potentially malignant disorders (OPMD), their terminology remains inconsistent and heterogeneous throughout the scientific literature, thus affecting evidence‐based decision‐making relevant for clinical management of these disorders. Updating this classification represents a necessity to improve the indexing and retrieval of OPMD publications, in particular for systematic reviews and meta‐analysis.

**Methods:**

Through a critical appraisal of the Medical Subject Headings (MeSH) and Excerpta Medica Tree (EMTREE) thesauri, we assessed gaps in the indexing for OPMD literature and propose improvements for enhanced categorisation and retrieval.

**Results:**

The present study identifies inconsistencies and limitations in the classification of these disorders across the major medical databases, which may be summarized in the following findings: a) The MeSH database lacks a dedicated subject heading for “oral potentially malignant disorders”; b) EMTREE indexing is incomplete, with only 5 out of 11 recognised OPMD having corresponding terms; c) Incoherent controlled vocabulary mappings hinder systematic literature retrieval.

**Conclusion:**

To ensure accurate evidence synthesis, the authors recommend searching both PubMed and Embase for OPMD studies. Moreover, the use of Embase’s PubMed query translator and Large Language Models, such as ChatGPT, may lead to retrieval biases due to indexing discrepancies, posing challenges for early‐career researchers and students. We recommend introducing “oral potentially malignant disorders” as a standardised subject heading. Evidence‐based medicine underpins clinical decision support systems, which rely on standardised clinical coding for reliable health information. Enhanced medical ontologies will facilitate structured clinical coding, ensuring interoperability and improving clinical decision support systems.

## 1 Introduction

In recent years, an increasing number of studies has been published in the field of medicine. This is improving our understanding of different diseases and their treatment. However, nowadays health science professionals are faced with an increasing amount of data and a growing number of information, which makes it difficult to assess what is the most appropriate clinical decision. In addition, the results of each individual investigation are often insufficient to provide definitive clinical solutions [1]. Thus, evidence‐based medicine (EBM) was developed. EBM is defined as “the conscientious, explicit and judicious use of current best evidence in making decisions about the care of individual patients.” According to this definition, the scientific literature is organized into hierarchical strata based on study design. At the top, there are systematic reviews and meta‐analysis, which today provide the strongest evidence‐based information, giving a comprehensive overview of what exist on a topic [2].

In systematic reviews, a standardized and reproducible protocol is followed to select, extract, and critically synthesize all relevant data on a topic to answer a specific clinical question and to highlight gaps. Systematic reviews may be integrated by meta‐analysis. Meta‐analysis is a statistical procedure by which data from previous independent investigations are pooled to obtain a single weighted result [3]. While performing a systematic review may appear an easy task, different requirements must be met. The development of methods and guidelines has improved the standardization in carrying out and reporting systematic reviews and meta‐analyses. The Cochrane collaboration group [4] and preferred reporting items for systematic reviews and meta‐analyses (PRISMA) guidelines represent a landmark in this field [5]. Among the different tasks required to perform a systematic review, the identification of source information is of notable importance. All potentially relevant publications should be obtained by a defined search strategy, ideally finding a compromise between precision and recall; while an opposite relationship exists between these, researchers must face the required time and resources needed to examine the high number of listed records. The goal of comprehensive searches for systematic reviews is to find as many relevant and important studies as possible for inclusion in the review [6].

Studies are mostly located in databases. MEDLINE/PubMed, Scopus, Web of Science, Embase, and Cochrane CENTRAL are the most relevant databases used in medicine and dentistry. Due to the variations in the articles included and indexing methods among databases, no database can provide an accurate and complete list of all studies that meet the requirements of a systematic review. These variations have led to recommendations from PRISMA‐search to use multiple databases to maximize the likelihood of finding pertinent studies [7]. However, when creating a search strategy, it is important to find a middle ground between the goal of comprehensiveness and preserving relevance. There are external factors that may prevent the retrieval of all relevant records. Two of the most important are incorrect indexing and inconsistent terminology. In this sense, the vocabulary used to conduct literature searches in the health sciences should represent the multidisciplinary nature of the topic. On the other hand, terms should be improved and updated as the science on a topic develops and evolves [8].

Thesaurus is defined as “a controlled vocabulary of organized arrangement of words and phrases used to index and/or to retrieve content through browsing or searching. It typically includes preferred and variant terms and has a defined scope or describes a specific domain” [9]. The most famous thesauri are Medical Subject Headings (MeSH) in MEDLINE/PubMed and Excerpta Medica Tree (EMTREE) in Embase.

## 2 Oral Potentially Malignant Disorders: An Evolutionary Concept

“Oral potentially malignant disorders” (OPMD) encompasses a group of heterogeneous diseases, affecting the oral cavity, with an increased risk of developing oral squamous cell carcinoma (OSCC) [10]. The worldwide prevalence is estimated to be 4.47% with rates up to 10.54% depending on the disorder and study population [11, 12]. This terminology was firstly introduced in the 2005 consensus report by the WHO Collaborating Centre for Oral Cancer and Precancer to replace the terms of “*precancer*,” “*epithelial precursor lesions*,” “*premalignant*,” “*precancerous lesions* or *conditions*,” and “*intra‐epithelial lesion*” [13]. Recently, in 2020, the OPMD list was updated. The OPMD list encompasses 11 different disorders, including immune disorders, tobacco‐related lesions, and other disorders of uncertain or unknown etiology. This list has undergone different updates over the years as the advancement of knowledge about OPMD helped in part to understand their natural history and predisposition to OSCC; however, with uncertainty [14]. These terminologies employed to characterize OPMD and listed diseases were retrieved from the original publications of consensus reports respectively in 1997 [15], 2007 [13], 2017 [16], and 2020 [17] and can be seen in Table 1. Updating this classification represents a necessity for clinicians to make evidence‐based decisions regarding the treatment of patients diagnosed with these disorders. Hence, systematic reviews and meta‐analysis represent an important step forward in these objectives. However, the heterogeneity in the different editions of the OPMD classifications, the disorders included, and their terminology could lead to shortcomings and pitfalls when conducting a systematic review. Particularly when addressing prevalence or outcomes of different interventions.

**TABLE 1:** Terminology of OPMD released from 1997 to 2020 Consensus reports.

1997		2007	2017	2021
***Synonyms***	Precancer			**Notes:** *Limited evidence to include **epidermolysis bullosa**, **chronic hyperplastic candidiasis** and **exophytic verrucous hyperplasia** within OPMD. Therefore, the above‐mentioned disorders are not included. **Fanconi anemia** and **Plummer‐Vinson syndrome** are listed as genetic syndromes associated to a higher risk of developing OSCC*
Epithelial precursor lesions				
Premalignant				
Intra‐epithelial neoplasia				
***Precancerous lesions***		***Oral potentially malignant disorders***		
Leukoplakia		Leukoplakia	Leukoplakia	Leukoplakia
Erythroplakia		Erythroplakia	Erythroplakia	Erythroplakia
Palatal lesions in reverse smokers		Palatal lesions in reverse smokers	Palatal lesions in reverse smokers	Palatal lesions in reverse smokers
***Precancerous conditions***				
Lichen planus		Lichen planus	Lichen planus	Oral Lichen planus
Oral submucous fibrosis		Oral submucous fibrosis	Oral submucous fibrosis	Oral submucous fibrosis
Discoid lupus erythematosus		Actinic keratosis	Actinic keratosis (lip only)	Actinic keratosis/actinic cheilitis
Epidermolysis bullosa		Discoid lupus erythematosus	Discoid lupus erythematosus	Oral Lupus Erythematosus
Xeroderma pigmentosum		Epidermolysis bullosa	Proliferative verrucous leukoplakia	Proliferative verrucous leukoplakia
Sideropenic dysphagia		Dyskeratosis congenita	Dyskeratosis congenita	Dyskeratosis congenita
Syphilis			Chronic candidiasis	Oral lichenoid lesion
			Syphilitic glossitis	Oral graft versus host disease
			Smokeless tobacco keratosis	
			Erythroleukoplakia	

## 3 Drawbacks and Heterogeneity in MeSH and EMTREE Thesauri for OPMD

When approaching a systematic review on OPMD, the first thing to do would be to create the most appropriate search strategy by examining the available MeSH terms. When the MeSH term “oral potentially malignant disorder” is queried, the search returns 0 results. The results are even worse if “OPMD” is used, resulting in the following MeSH “OpmD protein, *Pseudomonas aeruginosa* [Supplementary Concept],” for which 2 results are retrieved when searching PubMed.

When looking at the 11 specific OPMD, these are matched by a MeSH term in 8 cases, with ambiguity and more general terminology. This is the case of oral lupus erythematosus, matched by both “Lupus Erythematosus, Systemic” and “Lupus Erythematosus, Discoid” MeSH terms. Oral graft versus host disease is covered by the broad MeSH of “graft versus host reaction.” “Proliferative verrucous leukoplakia,” “palatal lesions in reverse smokers,” and “oral lichenoid lesions” lack of a MeSH term and thus indexing. When it comes to indexing, only “oral lichen planus,” “oral submucous fibrosis,” and “actinic cheilitis” are indexed as “mouth diseases.” “Leukoplakia,” “erythroplakia,” and “actinic keratosis” belong to “precancerous conditions.”

PubMed/MEDLINE MeSH indexing is still relying on the year 1997 terminology and the use of “precancerous conditions” nowadays, may be misleading, as this term includes 10 different conditions in the MeSH database, of which only 3 may be related to OPMD, such as “leukoplakia,” “erythroplakia,” and “actinic keratosis.” The other 7 terms listed are “aberrant crypt foci,” “Barrett esophagus,” “lymphomatoid granulomatosis,” “preleukemia,” “smoldering multiple myeloma,” “uterine cervical dysplasia,” and “xeroderma pigmentosum.” This leads to a decrease in precision of results when performing a systematic review, as the term “precancerous conditions” is not the same as OPMD. Nowadays, in a semantic point of view, the term “precancerous” should not be used to address patients with OPMD because not all the OPMD patients will develop OSCC.

About EMTREE, the indexed term “oral potentially malignant disorder” was added back in 2021. However, only 5 terms are listed and included: “actinic cheilitis,” “oral erythroplakia,” “oral leukoplakia,” “oral lichen planus,” and “oral submucous fibrosis.” Of interest, “oral leukoplakia” includes the terms “chronic hyperplastic candidiasis,” “proliferative verrucous leukoplakia,” “erythroleukoplakia,” and “hairy leukoplakia.” It should be noted that nowadays “chronic hyperplastic candidiasis” and “hairy leukoplakia” are not considered OPMD; “erythroleukoplakia” is a type of non‐homogeneous leukoplakia, and “proliferative verrucous leukoplakia” is considered an OPMD other than leukoplakia. Therefore, these EMTREE terms should be thoroughly reviewed. “Proliferative verrucous leukoplakia” should be indexed apart under “oral potentially malignant disorder” as being separate conditions. About erythroleukoplakia, WHO Consensus report classifies erythroleukoplakia as a non‐homogeneous leukoplakia, and it is acceptable being indexed under “oral leukoplakia.” However, “hairy leukoplakia” should be removed as does not belong to OPMD, and there is no evidence of increased risk of developing cancer in the areas affected by this condition.

Overall, EMTREE provides a better indexing of OPMD, however in this thesaurus, the item “palatal lesions in reverse smokers” is not identified.

In the last instance, “actinic keratosis/cheilitis” requires further discussion. WHO Consensus report identified “actinic keratosis” and “actinic cheilitis” as synonyms. In this case, we encourage the use of “actinic cheilitis” and therefore the indexing under both MeSH and EMTREE terms of “actinic cheilitis.” While “actinic keratosis” may affect the skin and the lips, “actinic cheilitis” is specific of the lips. Referring to chronic sun exposure damage of the lips, “actinic cheilitis” should be the terminology of choice and indexing, expecting better precision of results during systematic reviews. Indeed, for the EMTREE indexing, “actinic cheilitis” belongs to both “oral potentially malignant disorder” and “actinic keratosis.” This correctly summarizes the disease classification, physiopathology, histopathological characteristics, and its developmental nature. However, MeSH indexing requires improvements; “actinic cheilitis” is listed as supplementary concept, mapped to “cheilitis” and indexed as “lip diseases” However, this includes a wide range of lip conditions due to different factors. On the other side, “actinic keratosis” is indexed as “precancerous conditions,” with consequently limitations and confusion already discussed above.

All the MeSH and EMTREE keywords and relative indexing with their respective results in PubMed and Embase databases are summarized in Table 2. Please notice the relative categorizations and the distinct number of references retrieved.

**TABLE 2:** MeSH and EMTREE terms with related subject indexing headings and retrieved results, only from OPMD listed in the 2021 WHO Consensus report (numbers refer to the search performed in PubMed and Embase databases on 28th February 2024).

2021	MeSH terms	Results PubMed	Subject heading in MeSH	EMTREE terms	Subject heading in EMTREE	Results*/syn* Embase
**Notes:** *Limited evidence not included among OPMD 1. Epidermolysis bullosa 2. Chronic hyperplastic candidosis 3. Oral exophytic verrucous hyperplasia*	1. Epidermolysis bullosa 2. Candidiasis, chronic mucocutaneous 3. Focal epithelial hyperplasia	1. 5711 2. 654 3. 179	1. Skin abnormalities 2. Candidiasis 3. Mouth diseases	1. Epidermolysis bullosa 2. Chronic hyperplastic candidiasis 3. Verrucous hyperplasia	1. Congenital skin disease 2. Oral leukoplakia	1. 11 230 2. 95 3. 236
Oral potentially malignant disorders	N/A	N/A	N/A	Oral potentially malignant disorder	Mouth disease	2574
Leukoplakia	Leukoplakia	5594	Precancerous Conditions	Oral leukoplakia	Oral potentially malignant disorder	2696
Erythroplakia	Erythroplakia	486	Precancerous Conditions	Oral erythroplakia	Oral potentially malignant disorder	70
Palatal lesions in reverse smokers	N/A	N/A	N/A	N/A	N/A	N/A
Oral lichen planus	Lichen Planus, Oral	2896	Mouth Diseases	Oral lichen planus	Oral potentially malignant disorder	4143
Oral submucous fibrosis	Oral Submucous Fibrosis	1093	Mouth Diseases	Oral submucous fibrosis	Oral potentially malignant disorder	1730
Actinic keratosis (Actinic cheilitis)	1. Keratosis, Actinic 2. Cheilitis/Actinic cheilitis [Supplementary concept]	1. 2575 2. 1477/159	1. Precancerous conditions 2. Mouth diseases	1. Actinic keratosis 2. Actinic cheilitis	1. Skin disease 2. Oral potentially malignant disorder	1. 10 332 2. 568
Oral lupus erythematosus	1. Lupus erythematosus, systemic 2. Lupus erythematosus, discoid	1. 68 336 2. 3434	Connective tissue diseases	Lupus erythematosus	Skin disease	157 171
Proliferative verrucous leukoplakia	N/A	N/A	N/A	Proliferative verrucous leukoplakia	Oral leukoplakia	263
Dyskeratosis congenita	Dyskeratosis congenita	760	Skin diseases, genetic	Dyskeratosis congenita	Skin disease	2378
Oral lichenoid lesion*	N/A	N/A	N/A	1. Oral lichenoid reaction 2. Oral lichenoid lesion		1. 81 2. 91
Oral graft versus host disease	Graft versus host reaction	6761	Transplantation immunology	Graft versus host reaction	Immunopathology	94 235
Fanconi anemia	Fanconi anemia	3568	Anemia, hypoplastic, congenital	Fanconi anemia	Inherited cancer‐predisposing syndrome	11 487
Plummer‐vinson syndrome	Plummer‐vinson syndrome	259	Esophageal motility disorders	Plummer vinson syndrome	Dysphagia	438

*Note:* *planned in next EMTREE release.

## 4 What Can Be Done Now? Future Directions

As we have discussed throughout this manuscript, there is a need to update the indexing of the MeSH and EMTREE thesauri to improve the overall classification and retrieval of published articles on OPMD. Therefore, the agencies involved in these tasks should implement these changes. This will allow more appropriate and accurate bibliographic searches to be performed. This will result in a higher quality of the studies and a reduction in the time required for screening references.

National Library of Medicine has announced the New MeSH Descriptors for the 2025. However, no reference to OPMD is available. Similarly, the EMTREE release announcement for 2024 regarding the addition of new terminology does not include reference to the latest updated OPMD classification. However, for “oral lichenoid lesion” two terms are proposed: “oral lichenoid reaction” and “oral lichenoid lesion.” The WHO Consensus report on OPMD, includes both terminologies, differentiating from “oral lichen planus.” We suggest the inclusion of both terminologies as synonyms, since the published articles may include differently “reaction” or “lesion,” referring to the same OPMD, “oral lichenoid lesion.”

Another point to discuss is the release by Embase of the PubMed query translator tool. In its *beta* version, it allows the translation of PubMed syntax, including MeSH, into Embase. Of course, this gives rise to further research, as most of the EMTREE terms are not correctly matched and indexed in the MeSH database. So, the accuracy of the translations and thus the results of the studies retrieved when searching need to be addressed in future studies.

Nevertheless, in the last instance, the impact of controversies over large‐language models should not be underestimated. Scientists, clinicians, or students could access these models for consultation. If you ask ChatGPT 3.5 to list the MeSH terms for OPMD, the results will indicate “precancerous conditions” [Mesh] among its keywords, as well as “mouth neoplasms” [MeSH]. The use of these entry terms will result in a high number of studies, which are difficult to screen, since 10 subdomains are indexed under “Precancerous Conditions” of which only 3 may be referred to the last OPMD classification.

Moreover, by asking ChatGPT to match OPMD to MeSH or EMTREE, these models may fail. So, for example when asking: “Imagine being a systematic review expert—Could you provide me with a tabular list of MeSH terms for PubMed and EMTREE terms for Embase for a search on oral potentially malignant disorders?”. In this scenario, OPMD are matched by “mouth neoplasms” [MeSH] and “oral mucosa lesion”/exp. for EMTREE. These failures were already noted in a recent short communication published by Diniz‐Freitas et al. [18]. These authors highlighted that ChatGPT response to “What oral conditions are considered OPMD?” included only the following disorders: leukoplakia, erythroplakia, oral submucous fibrosis, lichen planus and actinic cheilitis; which differs from the 11 disorders in the last WHO Consensus report.

Finally, it is important to emphasize that EBM remains at the foundation of any clinical decision support system. EBM requires the use of standardized clinical coding apparatus to foster the future development of reliable health information systems [19]. They enable the uniform and trackable progression and evolution of pathognomonic entities, such as OPMD. In fact, well‐defined medical ontologies (which can integrate diverse sources of medical evidence, including clinical trials, guidelines, and patient records) provide a standardized framework for representing medical knowledge, including diseases, symptoms, treatments, and outcomes. This standardization ensures that clinical decision models are based on consistent and reliable data, which is a core principle of EBM and further improves data interoperability and the development and enhancement of decision algorithms, further fostering the offer of personalized recommendations.

## 5 Conclusion

It is necessary to update the MeSH and EMTREE thesauri indexing to improve the overall classification and retrieval of published articles on OPMD, based on the latest update. We suggest combining MeSH terms with free‐text keywords, depending on the specific objectives of the studies dealing with general or more specific OPMD. Caution should be taken when employing the PubMed to Embase search translational tool, as no precision studies have been performed and controversies exist between these two databases. We encourage searching both PubMed and Embase. Students and inexperienced researchers should refrain from using large‐language models when conducting a systematic review on OPMD.

## Author Contributions

Conceptualization: V.C.A.C., R.M.L.‐P. Data curation: V.C.A.C., R.M.L.‐P. Formal analysis: V.C.A.C., R.M.L.‐P. Funding acquisition: R.A.M., L.L.M., R.M.L.‐P. Investigation: V.C.A.C., R.M.L.‐P., G.M., M.P.S., L.L.M., R.A.M. Methodology: V.C.A.C., R.M.L.‐P. Project administration: L.L.M., R.A.M. Resources: L.L.M., R.A.M. Software: V.C.A.C., R.M.L.‐P. Supervision: V.C.A.C., R.M.L.‐P. Validation: G.M., M.P.S., L.L.M. Visualization: V.C.A.C., R.M.L.‐P., G.M., M.P.S., L.L.M., R.A.M. Writing – original draft: V.C.A.C., R.M.L.‐P., G.M., M.P.S., L.L.M., R.A.M. Writing – review and editing: V.C.A.C., R.M.L.‐P., G.M., M.P.S., L.L.M., R.A.M. All the authors contributed to the writing and revising the draft. All the authors approved the final version of this manuscript. MeSH and EMTREE thesauri were accessed.

## Ethics Statement

The authors have nothing to report.

## Consent

The authors have nothing to report.

## Conflicts of Interest

The authors declare no conflicts of interest.

### Peer Review

The peer review history for this article is available at https://www.webofscience.com/api/gateway/wos/peer‐review/10.1111/jop.13616.

## Data Availability

Data are available on MeSH and EMTREE databases.
